# Establishment of a multiplex PCR-CE assay for the simultaneous and rapid analysis of age markers for *Calliphora vicina* pupae

**DOI:** 10.1007/s00414-023-03013-1

**Published:** 2023-05-24

**Authors:** Katharina Hartmann, Malte Bamberg, Sandra Seum, Jens Amendt, Marcel A. Verhoff, Richard Zehner

**Affiliations:** 1 Institute of Legal Medicine, University Hospital Frankfurt, Goethe University, Frankfurt Am Main, Germany; 2 Department of Aquatic Ecotoxicology, FB Biowissenschaften, Goethe University, Frankfurt Am Main, Germany

**Keywords:** Forensic entomology, Blow fly, Age estimation, Age prediction tool, Postmortem interval

## Abstract

Insects, especially blow flies, are forensically relevant to determine the minimal postmortem interval (PMI_min_), based on the fact that they are usually the first colonisers of a body. By estimating the age of immature blow flies, interferences can be made about the time since death. Whilst morphological parameters are valuable for age estimation of blow fly larvae, gene expression profiling is more applicable for blow fly pupae. Here, the age-dependent changes in the gene expression levels during the development are analysed. 28 temperature-independent markers have already been described for the age estimation of pupae of the forensically important blow fly *Calliphora vicina* and are analysed by RT-qPCR. To allow simultaneous analysis of these age markers, a multiplex assay was developed in the present study. After reverse transcription, the markers are analysed simultaneously in an endpoint PCR and subsequently separated by capillary electrophoresis (CE). This method is highly attractive due to its quick and easy procedure and interpretation. The present age prediction tool was adapted and validated. The multiplex PCR assay reproduced the same expression profiles as the RT-qPCR assay based on the same markers. The statistical evaluation shows that the new assay has a lower precision but a better trueness for age determination compared to the RT-qPCR assay. Since the new assay is also qualified to estimate the age of *C. vicina* pupae and is practical, cost-effective and, even more importantly, time-saving, it is attractive for use in forensic casework.

**Supplementary Information:**

The online version contains supplementary material available at 10.1007/s00414-023-03013-1.

## Introduction

Necrophagous insects are forensically relevant indicators to restrict the minimum postmortem interval (PMI_min_). Blow flies are mainly used for this, as they are very often the first colonisers on a corpse. Age estimation of the oldest developmental stage provides the most valuable PMI_min_ [1, 2]. To determine the age of the immature blow flies, various parameters such as development rate and morphological changes like length or weight can be used. By taking into account the ambient temperature and species-specific growth rate, a precise age estimation can be possible, depending on the quality of the reference data [3–6].

Considering that the pupal stage of blow flies is about 50% of the juvenile development [5], an accurate method for age determination of pupae is constructive. However, age estimation of pupae is more difficult compared to the age estimation of larvae due to the lack of observable morphological changes without a complicated preparation of the puparium or a time-consuming further rearing up to the adult fly [7]. During metamorphosis, many different developmental processes occur, in which a large number of differentially expressed genes are involved. The analysis of the activity of such genes can be valuable to predict pupal age. Genetic changes may be more objective than morphological changes, making this method more attractive. Quantitative reverse transcription PCR (RT-qPCR) is the most common method to quantify the expression levels of differentially expressed genes. Several molecular age markers have been described to analyse the expression of candidate genes for different fly species [8–11].

Zajac et al. [12] have described molecular age markers for 15 different developmental stages of pupae of the blow fly *Calliphora vicina* Robineau-Desvoidy 1830, which have a faster change in gene expression during metamorphosis than observed for other age-dependent transcripts described in the literature [8–11]. The validation of the resulting qPCR assays for each of these age markers, labelled A1/A2–O1/O2, has been performed for three different constant breeding temperatures (17 °C, 20 °C, and 25 °C) covering a common forensic temperature range [12]. Each marker is maximally expressed at a certain stage of pupal development. No temperature dependence of the performance of the markers during metamorphosis was observed, except for one. The consecutive study investigated the functionality of the markers at different temperature conditions (constant or fluctuating). At the same mean temperature but at different temperature conditions (constant 10 °C vs. fluctuating 5–15 °C and constant 20 °C vs. fluctuating 15–25 °C), the marker genes show uniform expression patterns during metamorphosis. Additionally, a statistical tool has been developed in the R programming language, which enables age estimation based on the comparison of a certain gene expression pattern with the patterns of all age markers. Two reference databases for the age prediction tool have been established. The prediction quality of the validation of the tool demonstrated a mean absolute deviation (MAD) of 15.12% development and a root mean square error (RMSE) of 15.23% development. Thereby, estimation of the age of a *C. vicina* pupa based on the analysed gene expression data is possible [13].

Despite the fact that RT-qPCR is quantitative and highly sensitive and no post-amplification processing is required, the limited number of the available dye channels of the qPCR-system complicates or prevents a sound multiplexing. Hence, an assay consisting of endpoint PCR and capillary electrophoresis (CE) with upstream reverse transcription was developed. This technology enables the concurrent analysis of several or perhaps all molecular age markers in one reaction due to multiplex endpoint PCR. The simultaneously amplified age markers are fluorescence labelled and will be separated and detected based on size by capillary electrophoresis. In addition to the enormous time saving due to the simultaneous analysis of the age markers, the low cost and small amount of starting material required also make this technique attractive. Furthermore, the expression status is only measured qualitatively or semi-quantitatively. Therefore, the analysis of the reference genes can be omitted because no normalisation is required. This simplifies the data analysis.

For the present study, the previously investigated markers by Zajac et al. [12] and Hartmann et al. [13] have been used to develop a multiplex assay for age estimation of *C. vicina* pupae. Pupae bred under the breeding conditions as previously described were examined: constant temperatures of 10 °C and 20 °C, as well as uniformly fluctuating temperatures with corresponding mean values, i.e. 5–15 °C and 15–25 °C. The new age estimation system is supposed to include a reverse transcription followed by a multiplex endpoint PCR and a subsequent capillary electrophoretic separation for the simultaneous analysis of the aforementioned molecular age markers (in total 28). In addition, the age prediction tool has been adapted and rewritten to statistically evaluate the data collected by the multiplex assay. A blind study with gene expression data of *C. vicina* pupae bred outdoors was used for validation.

## Materials and methods

*Calliphora vicina* breeding, RNA isolation and quantification were performed as described in Hartmann et al. [13].

### Breeding and sampling

Established stocks of *C. vicina* at the Institute of Legal Medicine in Frankfurt am Main, Germany, were used for this study. After oviposition, for which the flies were provided with a piece of pig liver for 3 h, the eggs were incubated at 25 °C ± 1 °C for 24 h. After hatching, groups of 40 larvae were transferred to 40 g minced meat (50% pork/50% beef) and were bred at four different breeding conditions: constant temperatures of 10 °C (CV10) and 20 °C (CV20), as well as fluctuating temperatures of 5–15 °C (CV5–15) and 15–25 °C (CV15–25) without light, respectively, temperature tolerance was ± 1 °C. The fluctuating temperature cycle was as follows: staying at the lower temperature for 9 h, increasing to the higher temperature within 3 h, staying at this temperature for 9 h and decreasing to the lower temperature within 3 h. In addition to the four different temperature profiles mentioned above, an outdoor breeding (CVO) was carried out to validate the adapted age prediction tool. The temperature range for this breed was between 8 and 27 °C with a mean of 15 °C. The exact temperature profile is given in Hartmann et al. [13]. Once pupation began, these pupae were culled until the day when most of the larvae had pupated. This day was the start of sampling and preparation: five pupae of every breeding were collected every 24 h (48 h for CVO), homogenised in 500 μl Trizol (TRI Reagent®, Sigma-Aldrich, Merck KGaA, Darmstadt, Germany) and stored until further processing at − 20 °C. A total of 330 *C. vicina* pupae were analysed. Sampling was finished with the eclosion of the first adult fly in the respective breeding series. Due to the different total development time required for each breeding condition, a different number of pupae have been analysed (counted days from oviposition to hatch): CV10: *n* = 105 (69 d), CV5–15: *n* = 95 (59 d), CV20: *n* = 60 (21 d), CV15–25: *n* = 70 (20 d).

### RNA isolation and RNA quantification

After homogenisation of the pupa, total RNA isolation was performed according to the TRI Reagent® Protocol (Sigma-Aldrich, Merck KGaA, Darmstadt, Germany). The RNA pellet was dried for 5–10 min at 50 °C and subsequently dissolved in RNA Storage Solution (Thermo Fisher Scientific, Waltham, USA) and stored at − 20 °C. Possible co-extracted DNA was removed by DNA digestion and verified and visualised by in-house PCR and gel electrophoresis. Quantification of total RNA was performed with the NanoDrop™ 1000 Spectrophotometer (Thermo Fisher Scientific).

### cDNA synthesis

The reverse transcription was performed with 0.1 μg/μl of total RNA. cDNA was synthesised using the High-Capacity cDNA Reverse Transcription Kit (Thermo Fisher Scientific) according to the manufacturer’s protocol with the exception of using 4 U DNase instead of 2 U DNase.

### Endpoint PCR-based gene expression profiling

Age-dependent gene expression of *C. vicina* pupae was analysed by amplification of the validated molecular age markers except markers E1 and E2 [12]. At first, the markers were amplified using singleplex PCR for establishment of the new assay. The singleplex PCR was performed using 0.5 μl cDNA and the AmpliTaq Gold DNA Polymerase with buffer I (Thermo Fisher Scientific) according to the manufacturer’s protocol and a final concentration of 0.2 mM dNTP Mix in a total reaction volume of 25 μl. First of all, a primer concentration of each 0.2 μM was used. The following cycling conditions were applied for each singleplex PCR: 95 °C for 10 min, 26 cycles of 95 °C for 30 s, 60 °C for 30 s, 72 °C for 45 s and a final extension at 72 °C for 30 min. After successful analysis of the age markers, the primer concentrations were optimised and two different multiplex PCRs were developed (Table 1). For both multiplex PCRs, the initial denaturation was also at 95 °C for 10 min, followed by 31 (PCR 1) and 29 (PCR 2) cycles of 95 °C for 30 s, 60 °C for 30 s, 72 °C for 45 s and a final extension at 72 °C for 30 min.

**Table 1:** Primer characteristics used for age marker amplification in the two multiplex PCRs. Each marker is maximally expressed at a certain stage of pupal development. Primer sequences of D1 and D2 according to [13], all others according to [12]

Marker	Sequence (5′ → 3′)	Dye	Size (bp)	Final primer concentration (μM)
A1^b^	f: CTCCTGCCATCTGTGTTTCACT r: GAGGGAGACGTATAGTAATGGCAG	ROX	139	0.2
A2^a^	f: GCAAAGATGCCGAGAATCCCA r: TCATCAGCGTAACTCACTCGTC	JOE	159	0.2
B1^a^	f: ACATCTCCGCTCGCATTCTCC r: CGTGTAACCAAGCTCCGCATT	ROX	163	0.2
B2^a^	f: TCTTGGGTGCAGGACGACATT r: GGGTAGACTCTCGTCGTTGTG	6-FAM	66	0.2
C1^a^	f: CCAGCTGCCGTTACTCCTTATC r: CCGTAGACTTCATCGGGTTGTT	JOE	100	0.06
C2^b^	f: GATGAAGTCTACGGCCCACC r: CTGCGAGCAGATGAAGTACGG	Cy3	189	0.2
D1^b^	f: AATCGTGGGGATGTGGCAAA r: TGTACCCACTGCTTTCACCG	JOE	144	0.2
D2^b^	f: AGGCAGCAGTAGTGTCGAAT r: CCGTCAAGCTCGTTTGGCTA	ROX	174	0.2
F1^b^	f: GGCCTTAAGCTCTAATTGTCCCTC r: CTTGATATTGCCGGAGCCCA	6-FAM	160	0.06
F2^a^	f: AGGACAGTTGATGTCCGGTTTC r: GCTAGACATGGTGTGAATTCGGG	JOE	125	0.2
G1^a^	f: ACGTTGACAAGTGTCTGGCTC r: CTGGCTATGACGCTCTCGCA	ROX	179	0.2
G2^b^	f: AGCCCAATACGAAGGAGCCA r: CAGTCAGCCATCGTTCCATTCTTG	Cy3	159	0.2
H1^a^	f: GGGCTATTCTACACATCATACGGG r: ACCAAGACCGTGAGCCTGTT	ROX	115	0.33
H2^a^	f: CCGCCCTGATAGCAATTATAGTCC r: TCTGAGACTTAGTGCGCTGTCC	Cy3	173	0.2
I1^b^	f: AAGAAACGCTCTGGACGCAA r: CGAGCCAAGAATGGAGGTGG	6-FAM	102	0.06
I2^a^	f: GCGGTGCCCAACTACCAAATAA r: TACTGACACCACTTAGACCCGA	JOE	189	0.2
J1^b^	f: CCAGGAGGGCAAATGTAGACCA r: TCCATACCCACTGCCGTTTC	JOE	176	0.06
J2^b^	f: TCACCATCTCTGGTCTCCCAA r: GCTCATGAGGATTATGAGGGTGG	6-FAM	130	0.1
K1^a^	f: GGAGAAGACAGGACAGACTTGG r: CAAGCCGCCAAACAATACGG	6-FAM	148	0.1
K2^a^	f: CGAGTGGGTGGCAACAAGAA r: CCATACGCGAAGTTCCGACA	Cy3	167	0.2
L1^a^	f: TGTTGACACTGGCGAAGTGGA r: CTACGCTCGCCTTCTACATCATC	Cy3	144	0.2
L2^b^	f: CCGCTAGGAGCAGAAGGTAGT r: CCTCCATCAGGTGTAGGAAGTGA	6-FAM	195	0.2
M1^a^	f: ATTCACAAACCGGCAAGGGT r: GCCAGCAGTGTAGGAGCAAA	JOE	172	0.2
M2^a^	f: GCAACAATGGGAGCAGCAAC r: TTGACCGGTGACAGCAAGAG	6-FAM	176	0.06
N1^a^	f: GAGCAGCACAAGCCAATCTCT r: ACATAATAAGGACGCCACGCTC	ROX	156	0.33
N2^b^	f: AAACGAGCGGGTACAGCCA r: GGGTTCCTACTCCGTTGTAGATG	6-FAM	172	0.2
O1^b^	f: GCTTTGTGCTTGTTGGCTGTTG r: AGGCTGTGGTGTAGGGTGAAG	JOE	79	0.2
O2^a^	f: TGTAGGCAGCAGTGTAGGGA r: CGCTATTGTTGCCTTGGCTG	Cy3	97	0.2

^a^Multiplex PCR 1

^b^Multiplex PCR 2

### Capillary electrophoresis

The amplified PCR products were separated and detected by capillary electrophoresis (CE) on a 3130xl Genetic Analyser (Applied Biosystems). 1 μl of each PCR product was added to 12 μl Hi-Di Formamide (Thermo Fisher Scientific) and 0.25 μl Orange 500 DNA Size Standard (500 bp; Nimagen). The samples were denatured at 95 °C for 3 min. The following run module settings were used for the capillary electrophoresis: 12 s injection time, 1.2 kV injection voltage, 3300 s run time, 15 kV run voltage, filter set G5 (6-FAM, VIC, NED, PET, LIZ) and POP-6 Polymer (Thermo Fisher Scientific). Results were analysed using GeneScan 3.7 Analysis Software (Thermo Fisher Scientific), Genotyper Software (Thermo Fisher Scientific) and Genoproof 3.0.7 (qualitype GmbH) with in-house designed binset. The binset covers the fragment lengths of the analysed age markers. The threshold of the peak height for scoring age markers was set to 100 rfu (relative fluorescence units), i.e. by capillary electrophoresis, the presence or absence of a certain age marker can be detected. The electropherogram data were exported to GraphPad Prism 5.01 (GraphPad Software) and visualised in scatter plots. Every detected fluorescence signal of each age marker is subjected to the development of the respective examined pupa.

### Age prediction tool

The already described age prediction tool [13] was adapted (available on request) to evaluate the gene expression data collected in this study. The tool was developed using the statistical programming language R applying RStudio Version 1.1.456. In brief, a reference database is used to compare the analysed gene expression data of a pupa of unknown age. To generate this reference database, the collected gene expression data of each breeding were separately used and analysed. In addition, datasets of combined breeding were also evaluated: CVcold (includes CV10 and CV5–15), CVwarm (includes CV20 and CV15–25) and CVall (includes all four breeding). To validate the adapted age prediction tool, the measured gene expression data of *C. vicina* pupae of the outdoor breeding (CVO) were used and compared to the analysed reference databases.

## Results and discussion

**Fig. 1:**
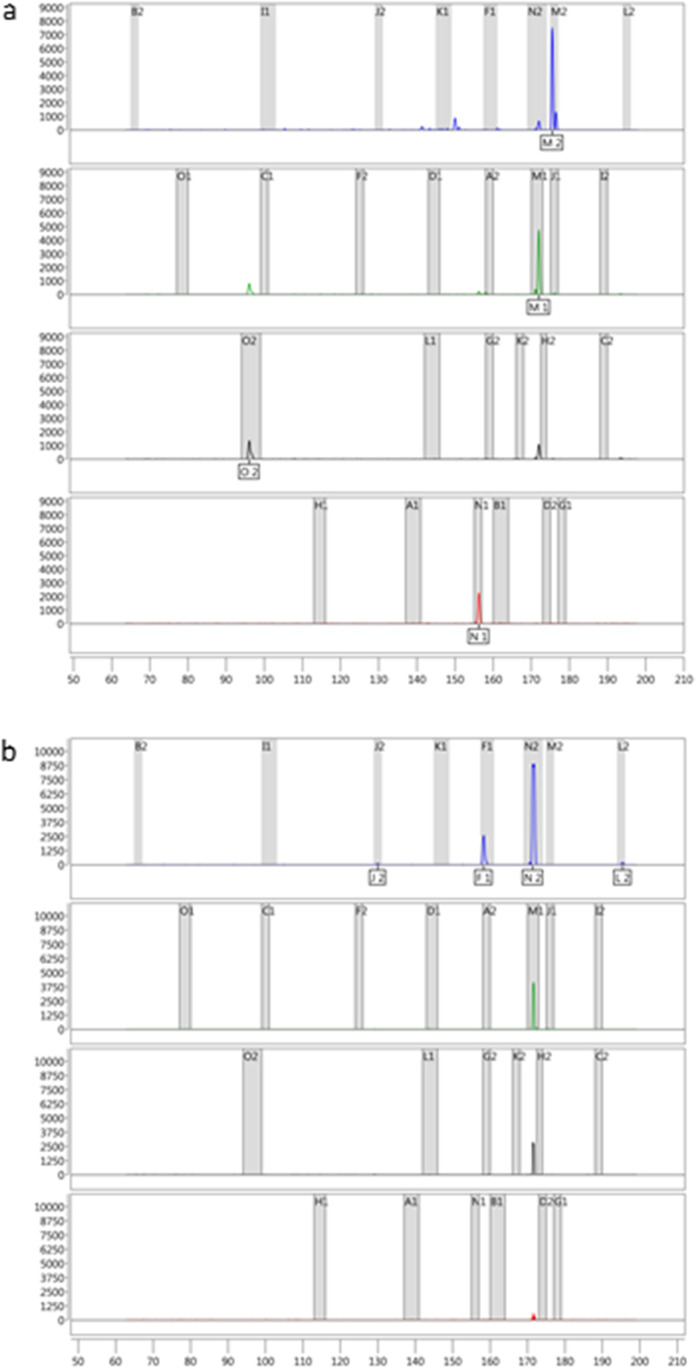
Gene expression profiles of a 90%-developed *C. vicina* pupa bred at fluctuating temperatures of 15–25 °C (CV15–25) analysed with the multiplex endpoint PCR assay. The electropherograms depict the results of **a** multiplex PCR1 and **b** multiplex PCR2. The binset (grey bars) indicates the fragment lengths (*x*-axis) of the age markers of both multiplex PCRs. The signal intensity (*y*-axis) of the peaks is given in rfu (relative fluorescence units)

Age estimation of necrophagous Diptera, such as Sarcophagidae or Calliphoridae, can help in the determination of the PMI_min_. In recent years, various genes have been described whose expression level allows conclusions about age [8, 10, 12, 14–16]. In this study, a multiplex assay for the simultaneous and rapid analysis of 28 recently described age markers [12, 13] was established for age estimation of *C. vicina* pupae. Two multiplex endpoint PCRs were developed: PCR1 encompasses 16 molecular age markers; PCR2 12 markers. The amplified PCR products were separated by capillary electrophoresis. The detected signals can be identified by a binset consisting of the fragment lengths of all molecular age markers. The results of the multiplex assay are presented in two electropherograms: one for PCR1 and one for PCR2. Figure 1 presents the results of the multiplex endpoint assay of a 90%-developed *C. vicina* pupa. The pupa analysed here was grown at fluctuating temperatures of 15–25 °C. Electropherogram a (Fig. 1a) shows the separation of the amplified age markers from PCR1 and electropherogram b (Fig. 1b) from PCR2. The markers M1, M2, N1 and O2 were detected by PCR1 and the markers F1, J2, L2 and N2 were detected by PCR2. For the markers M1, N2 and O2, “pull-up” or “bleed-through” peaks can be observed in some scenarios. These artefact peaks are caused when the signal intensity of the marker peak is too high due to unexpectedly high expression. A few markers are susceptible to pull-up peaks and have therefore been split accordingly in both multiplex PCRs to avoid false-positive detection, i.e. the pull-up peaks are either outside the binset or on bins of the other multiplex PCR. Thus, in this example, N2 shows a pull-up in the other colour channels (Fig. 1b). However, this has been taken into account when designing the multiplex assay: the pull-up peaks are located outside the binset or on the bin of marker M1 from PCR1 (marker N2 is in PCR2). An unequivocal interpretation is possible.

**Fig. 2:**
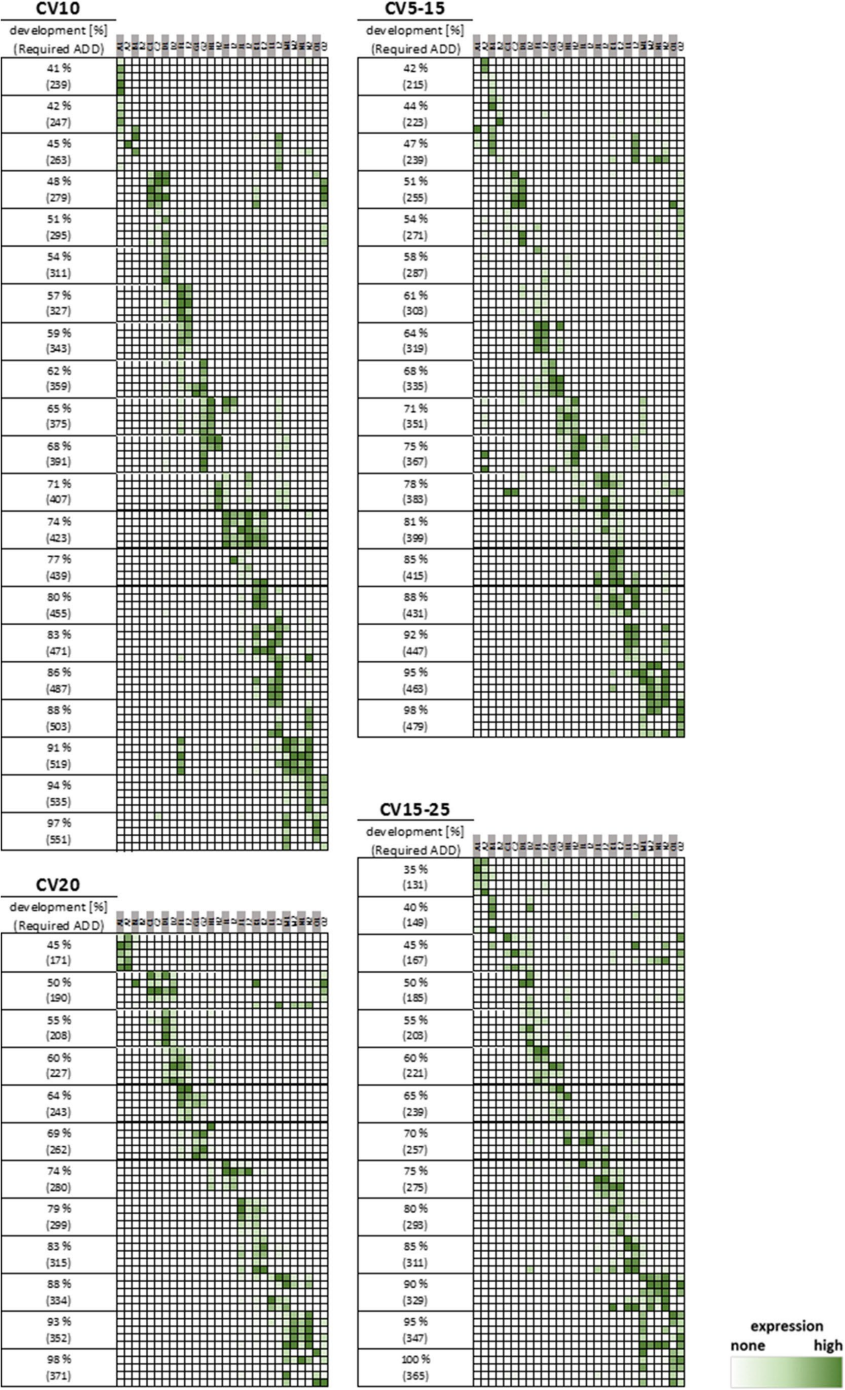
Heatmap of the marker expressions during pupal development of each *C. vicina* breeding (CV10, CV5–15, CV20, and CV15–25). 100% development corresponds to the complete development from oviposition to eclosion of the imago. The accumulation of a certain amount of temperature is required for the development and is given in accumulated degree days (ADD). The colour scale indicates the intensity of the relative fluorescence signal of each molecular age markers (represented in columns) of every analysed pupa (represented in rows according to development). The colour scale was set individually for each marker gene: dark green corresponds to the highest expression and white the lowest expression (= no detectable expression) of a certain marker during pupal development

The height of the peaks correlates with the amount of the amplified marker and consequently also with their expression levels within the dynamic range of the CCD (charge-coupled device) camera of the CE (capillary electrophoresis) instrument. Since the markers are expressed at different levels during pupal development, they show different peak heights in the electropherograms. Figure 2 illustrates the hierarchical clustering of the marker expressions of each *C. vicina* breeding (CV10, CV5–15, CV20 and CV15–25), revealing the specificity of marker expression during pupal development. Despite the dropout of individual markers, a characteristic expression of the age markers is evident in total. Thus, the early markers (starting at A1, A2, B1, etc.) have been detected in the early pupal phase and the late developmental markers (O2, O1, N2, etc.) are mainly expressed in the later pupal phase. It has also already been observed that some markers show a further expression peak during the pupal phase [12, 13]. Since the markers are expressed or not expressed at specific developmental stages, the expression data obtained by the multiplex assay can be used to determine the development stage of the pupa, allowing for an estimation of age in the end. The development stage of the examined *C. vicina* pupae is given in percentage time of development. Thus, the complete development from oviposition to eclosion of the imago corresponds to 100% development. This allows a better comparability of the data obtained at different temperature regimes [5, 13, 17].

The different markers show specific gene expression profiles during defined phases of the metamorphosis (Supplementary Information, Figure S1). The results of the gene expressions received by multiplex assay are consistent with the marker expression data of Zajac et al. [12] and Hartmann et al. [13]. So, at different constant and fluctuating temperature profiles, the expression of the marker genes behaves the same during metamorphosis of *C. vicina* pupae. But the marker performances are not equal. B2, D2, H2, I2 and N1 showed poorer performance. These markers dropped out during the analysis of particular breeding, and consequently, their gene expression could not be used for the subsequent validation of the age prediction tool. The age-dependent gene expression patterns are similar to the gene expression data obtained by qPCR [12, 13], regardless of the breeding conditions.

An existing statistical age prediction tool [13] has been adapted for the interpretation of the measured gene expression also by the multiplex assay. To predict the development (%), a reference database is necessary. For this, the gene expression data of each breeding collected in the present study were separately analysed and different databases were generated: CV10, CV5–15, CV20, CV15–25, CVcold (pooled data of the breeding at the colder temperatures CV10 and CV5–15), CVwarm (pooled data of the breeding at the warmer temperatures CV20 and CV15–25) and CVall (pooled data of all four breeding). The analysed expression of the age markers is given in rfu (relative fluorescence units). Consequently, the age prediction tool evaluates the rfu values of the individual markers. By importing the gene expression data into the age prediction tool, the 50%, 75% and 95% confidence intervals (CI) of each pupa for the seven databases were calculated (Table 2). These reveal that 50%, 75% and 95% of the data are within the bounds (given in % development) of the true age, respectively. The smaller the value, the smaller the CI, the smaller the deviation from the true age. The CI bounds show a wider range than those based on qPCR data for almost all of them [13]. This could be explained by the fact that the multiplex assay is just a semi-quantitative method. In contrast, the qPCR data are normalised against a reference gene, which may have a positive impact on the variance of the data. The data for CV5–15 are conspicuous. For the qPCR assay, smaller CI bounds were observed for this breeding compared to the other breeding, whereas wider CI bounds were observed for the multiplex endpoint PCR assay. This might indicate that the expression levels of the age markers in these *C. vicina* pupae (CV5–15) have a high variance, which can be corrected in the qPCR assay by normalisation. All in all, the multiplex assay shows a lower precision for age determination compared to the qPCR assay. However, it has to be pointed out that the multiplex PCR assay is far more practicable, cost-effective and, above all, time-saving.

**Table 2:** Bounds for the 50%, 75% and 95% confidence intervals of both assays (singleplex qPCR vs. multiplex endpoint PCR) according to the respective dataset (CV10, CV5–15, CV20, CV15–25, CVcold, CVwarm and CVall). The bounds are given in % development: 50%, 75% and 95% of the measurements are within the indicated distance to the true age

	Bound for 50% CI		Bound for 75% CI		Bound for 95% CI	
	qPCR	Endpoint PCR	qPCR	Endpoint PCR	qPCR	Endpoint PCR
CV10	0.65	0.37	1.36	1.65	4.04	6.80
CV5–15	0.06	0.77	0.41	2.10	2.97	6.62
CV20	0.28	0.49	1.22	1.24	4.53	5.61
CV15–25	0.52	0.67	1.48	1.25	4.32	4.37
CVcold	0.64	0.90	1.62	1.93	4.84	4.27
CVwarm	0.80	0.84	1.61	1.89	3.54	4.15
CVall	3.54	0.95	1.72	2.13	4.11	4.51

**Fig. 3:**
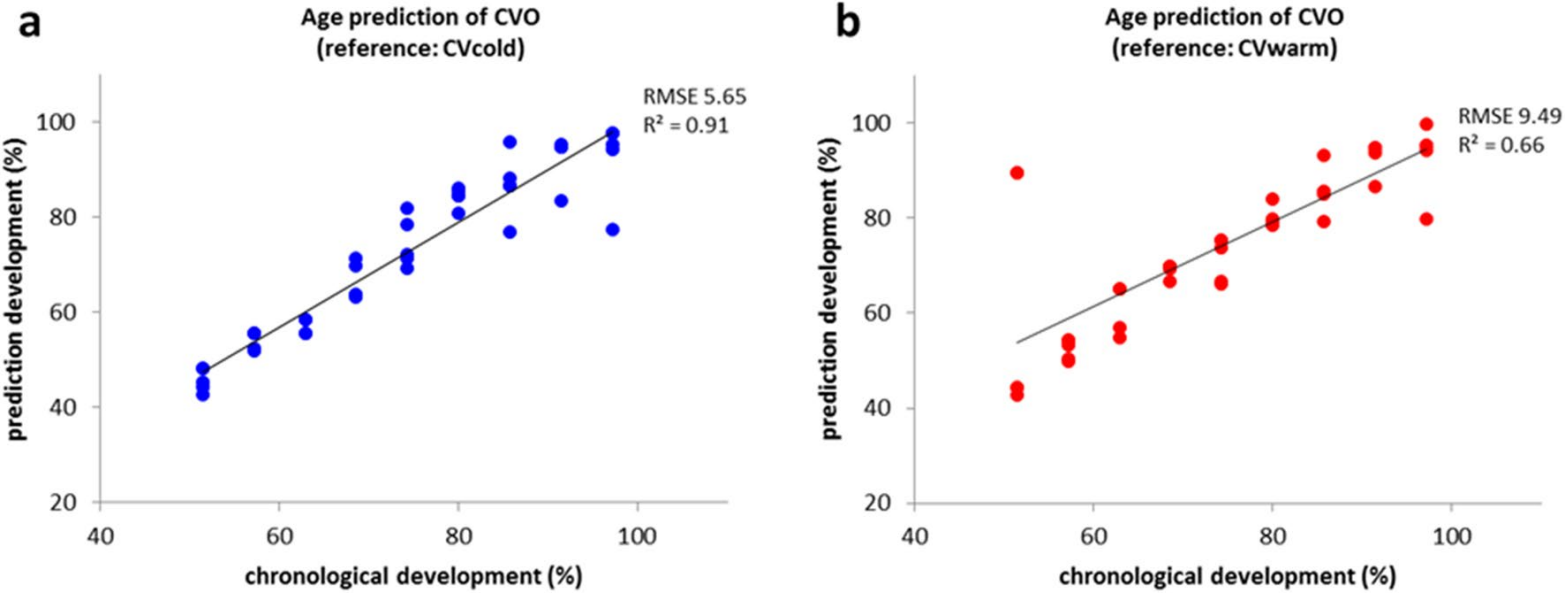
Validation of the age prediction tool: predicted vs. chronological development (%) of each *C. vicina* pupa of the outdoor breeding (CVO). The development stage was estimated using **a** CVcold and **b** CVwarm as reference data in the age prediction tool

In addition to precision, trueness was also analysed in order to finally evaluate the accuracy of the adapted age prediction tool. This validation was done with an outdoor breeding of *C. vicina* (CVO). The measured gene expression data of each *C. vicina* pupa of the outdoor breeding (CVO) were analysed and compared to evaluated reference databases. Since age estimation is only practicable within a defined temperature range which fits to the applied model [13], only CVcold and CVwarm were used as reference databases. For evaluation of the model performance, the RMSE (root mean square error) was calculated (Fig. 3). For the age prediction of the *C. vicina* pupae of the CVO, using CVcold as reference database, an RMSE of 5.65% development was obtained (Fig. 3a). For CVwarm as reference dataset, the RMSE value was 9.49% development (Fig. 3b). Accordingly, a better forecast quality for the outdoor collective is shown if CVcold is used as a reference. This is also supported by the coefficient of determination, which yields *R*^2^ = 0.91 for CVcold, whereas only *R*^2^ = 0.66 is achieved for CVwarm. Since the mean breeding temperature of CVO was 15.20 with a median of 15.06, which is straight in the middle between CVcold (mean temperature = 10 °C) and CVwarm (mean temperature = 20 °C), it initially has been assumed that a calculation with both reference datasets should have the same accuracy. However, this is disproved by our data showing a better accuracy and therefore a better quality for age prediction of *C. vicina* pupae of the CVO using CVcold as reference database. For the development of an insect, the accumulation of a certain amount of temperature is necessary. This summation of a specific amount of heat for development is given in accumulated degree hours or days (ADH or ADD). According to Greenberg and Kunich [18], the relation between rate of development and environmental temperature should be linear within a certain range. Thus, the amount of temperature necessary for complete development is constant for each insect species. Conversely, it has already been observed that colder temperatures have greater impact on the total development, implying that a higher amount of ADD is required for the complete development compared to breeding at higher temperatures [5, 17]. In our previous study, in which the gene expression of *C. vicina* pupae had been analysed by RT-qPCR, the effect of cold temperatures on the development was also observed [13]. There, a better RMSE value for % development was achieved for CVcold as reference database than for CVwarm. A comparison of the two assays (RT-qPCR vs. multiplex endpoint PCR) shows that the multiplex method in the outdoor breeding has demonstrated a better predictive power. Thus, despite a lower precision, a better trueness for age estimation of *C. vicina* pupae could be reached with the new assay.

## Conclusion

Analysis of the expression of age-related genes of *C. vicina* pupae can be used to estimate their age and subsequently the PMI_min_, which is important in forensic casework. A multiplex PCR-CE assay was established with which a simultaneous and thus rapid expression analysis of such age markers can be carried out. The multiplex assay consists of two multiplex endpoint PCRs in which the molecular age markers already described by Zajac et al. [12] and Hartmann et al. [13] are analysed. These previous studies applied RT-qPCR assays for expression analysis of the age markers. The gene expression profiles of the age markers received with the multiplex assay are consistent with the marker expression profiles obtained with the RT-qPCR assay. Since the accuracy of a method depends on both precision and trueness, it is not easy to define one of the two assays as the better one. Both methods, the RT-qPCR assay and the multiplex endpoint PCR assay, are practicable to estimate the age of a *C. vicina* pupa. Since the CI bounds of the reference databases CVcold and CVwarm show only low deviation between the two methods, the precision of the RT-qPCR assay and the multiplex endpoint PCR assay differs only slightly from each other. Due to this and the better trueness, as well as the more rapid analysis and less complex interpretation of gene expression data collected by multiplex PCR, the new multiplex endpoint PCR-CE assay is recommended for age determination of *C. vicina* pupae.

### Supplementary Information

Below is the link to the electronic supplementary material.

Supplementary file1 (DOCX 1204 KB)

## Data Availability

Not applicable.
