# Supplementary material for: Establishment of a multiplex PCR-CE assay for the simultaneous and rapid analysis of age markers for Calliphora vicina pupae
Source: Int J Legal Med. 2023 May 24;138(1):187–96. doi: 10.1007/s00414-023-03013-1 (PMC10771996; doi:10.1007/s00414-023-03013-1)
Supplement: Supplementary file 1 — Supplementary file1 (DOCX 1204 KB) [file 414_2023_3013_MOESM1_ESM.docx]

**Supplementary Figure 1:** Gene expression data of each marker of different *C. vicina* breeding. The relative gene expression (RFU: relative fluorescence units) during pupal development (%) are visualized in scatterplots. 100 % development corresponds to the complete development from oviposition to eclosion of imago.
